# R3J-AGNN: GNN-Based Prediction of Inter-Branch Angles in RNA Three-Way Junctions from Secondary Structure

**DOI:** 10.3390/biology15060457

**Published:** 2026-03-11

**Authors:** Hu Yang, Ning Qiao, Bengong Zhang, Ya-Zhou Shi, Ya-Lan Tan

**Affiliations:** 1 Research Center of Nonlinear Science, School of Mathematical and Physical Sciences, Wuhan Textile University, Wuhan 430200, China; 2 School of Biomedical Engineering and Health, Wuhan Textile University, Wuhan 430200, China

**Keywords:** RNA three-way junction, inter-branch angles, RNA structure prediction, graph neural network

## Abstract

Despite advances in computational RNA 3D structure prediction, accurately modeling multi-branch RNA motifs remains challenging, even with known secondary structures, because their global topology is highly sensitive to the relative orientations of the helical stems at the junction. Here, we propose R3J-AGNN, a dual-resolution hierarchical graph neural network that predicts inter-branch angles of RNA three-way junctions (3WJs) directly from secondary structure information. The model integrates fine-grained nucleotide-level interactions with a coarse-grained representation of the global topology of 3WJ-containing RNA structures, enabling accurate inference of inter-branch geometry and subsequent reconstruction of their 3D scaffolds. Evaluations on an independent test set demonstrate that R3J-AGNN achieves robust and consistent predictive performance. Thus, by inferring inter-branch angles of RNA three-way junctions from secondary structure information, R3J-AGNN provides actionable geometric constraints for RNA tertiary structure modeling and refinement.

## Simple Summary

Despite the essential role of three-dimensional RNA structures in cellular functions, accurately modeling the spatial organization of multi-branch junctions—particularly three-way junctions (3WJs)—remains a substantial challenge, even when the underlying secondary structure is known. Here, we introduce R3J-AGNN, a dual-resolution hierarchical graph neural network that predicts inter-branch angles of RNA 3WJs directly from secondary structure information. The model integrates fine-grained nucleotide-level interactions with a coarse-grained representation of global junction topology, enabling the inference of three-dimensional geometry from sequence-derived features alone. Evaluations on independent test sets demonstrate that R3J-AGNN achieves robust and consistent predictive performance across diverse junction architectures. By providing accurate geometric constraints for 3WJs, R3J-AGNN offers a practical tool for improving RNA tertiary structure modeling and refinement.

## 1. Introduction

RNA plays a central regulatory role in living systems, participating in gene expression, protein synthesis, and epigenetic control [1,2,3]. Its function is highly dependent on well-defined three-dimensional (3D) architectures [4,5,6,7], as exemplified by the ribosome, in which the precise 3D folding of ribosomal RNA (rRNA) forms the catalytic core that is essential for protein synthesis [8,9,10]. In many functional RNAs, multi-way junctions serve as key architectural hubs that determine the relative orientation of helical branches and mediate long-range tertiary interactions essential for global topology [11,12,13,14]. Among these, three-way junctions (3WJs) are the most prevalent type, formed by three helical branches converging at a central junction. Notably, the relative orientation of these branches—commonly described by inter-branch angles [15,16]—largely dictates the overall 3D conformation of 3WJs and, consequently, their functional capacity [17].

Despite the critical role of RNA structure, obtaining high-resolution structural information for RNA remains challenging. This is particularly true for complex motifs, such as multi-way junctions, whose inherent flexibility and thermodynamic instability [18] complicate crystallization and limit the interpretability of solution-state NMR ensembles [19,20]. Although recent advances in cryo-EM have enabled the visualization of large RNA assemblies [21,22], experimentally resolved RNA structures remain scarce. As of 1 December 2025, RNA-only structures constitute less than 3.0% of the Protein Data Bank (PDB) [23], and among these, structures with multi-way junctions represent an even smaller fraction.

Computational approaches offer a viable strategy to alleviate this data scarcity, yet accurately modeling multi-branch RNA motifs remains challenging even with known secondary structures. This is because their global topology is highly sensitive to the relative orientations of helical stems at the junction [9,24]. Traditional secondary structure prediction methods lack explicit 3D information and thus cannot resolve inter-helical orientations [25,26,27,28], while physics-based frameworks [29,30,31] often suffer from rugged energy landscapes that lead to inaccurately predicted junction topologies [32,33]. Even recent data-driven advancements face notable challenges in modeling multi-way junctions [34]. State-of-the-art deep learning methods, including structure predictors like DeepFoldRNA [35] and trRosettaRNA [36], as well as RNA evolutionary language models [37,38,39,40], often struggle to capture their native-like global arrangements [20,41]. This limitation stems from the fact that junction-region conformations depend not only on local sequence features but also on the intricate coupling between fine-grained nucleotide interactions and the coarse-grained topology of RNA structures [11,42].

To address these challenges, we propose a dual-resolution hierarchical graph neural network (GNN), named R3J-AGNN, specifically designed to predict inter-branch angles of 3WJs directly from secondary structure information. At the fine-grained (residue) level, a nucleotide graph is constructed with individual nucleotides as nodes and their pairwise interactions as edges. At the coarse-grained level, the RNA secondary structure is abstracted into a tree graph, in which loop regions—including junctions—serve as vertices, and helical stems form the connecting edges [43]. By integrating these two representations in an end-to-end framework, R3J-AGNN learns the relationship between residue-level interactions and global topology, enabling accurate inference of inter-branch geometry and subsequent reconstruction of 3WJ 3D scaffolds.

## 2. Materials and Methods

### 2.1. Tree-Graph Representation and Definition of Inter-Branch Angles

**Figure 1:**
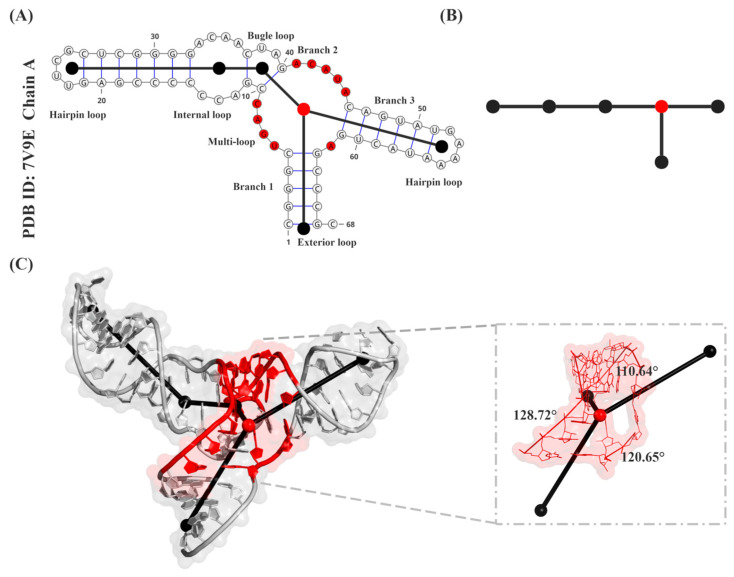
Multi-scale representation of an RNA three-way junction derived from RNA-Puzzles Target 34 (methyl transferase ribozyme, PDB ID: 7V9E, Chain A) [48]. (**A**) Secondary structure abstraction. The RNA secondary structure is abstracted into a tree graph, with loop regions modeled as nodes and connecting helical stems as edges. (**B**) Planar tree graph. This planar topological representation features the central three-way junction as a distinct red node, with all peripheral loops (nodes) and connecting stems (edges) depicted in black. (**C**) 3D tree graph. The corresponding tertiary structure is mapped onto a spatial 3D tree graph, where the junction region (red) and inter-branch angles are (black) prominently highlighted.

We adopt the planar tree-graph representation proposed by [43,44,45,46] to capture the overall topological information of RNA 3D structures. As illustrated in Figure 1A, the pseudoknot-free secondary structure of a representative RNA is abstracted by mapping all loop regions—including multi-way junctions—to nodes, with helical stems represented as edges connecting these nodes. The resulting planar tree graph (Figure 1B) provides a compact representation of the RNA’s topological organization.

To quantify the inter-branch angles in three-way junctions, we assign spatial coordinates to each node in the tree graph. Specifically, for standard nodes (i.e., those corresponding to non-junction regions), the coordinates are defined as the geometric centroid of the C4′ atoms of all nucleotides comprising the associated loop. For three-way junction nodes, coordinates are determined as the geometric incenter of a triangle formed by three anchor points. Each anchor is defined as the midpoint between the C4′ atoms of the closing base pair in the adjacent stem (Supplementary Figure S1).

We note that the geometric center of hydrogen-bonded base atoms could, in principle, provide a closer approximation to the helical axis of a regular RNA duplex [47]. However, closing base pairs at junction interfaces frequently exhibit structural fraying or adopt non-canonical geometries. To avoid introducing positional noise from such local distortions, we instead selected C4′ atoms as conformationally stable backbone anchors. This choice ensures robust extraction of global helical trajectories.

Using the assigned node coordinates, we compute the three inter-branch angles for each three-way junction; each angle is geometrically constrained to be less than 180°. To ensure consistency in angle assignment, the three stems of each junction are sequentially labeled as Branch 1, Branch 2, and Branch 3, following the 5′→3′ direction of the RNA chain. The inter-branch angles are then defined as $\theta_{1}$ (between Branches 1 and 2), $\theta_{2}$ (between Branches 2 and 3), and $\theta_{3}$ (between Branches 3 and 1). The angle set $\Theta \;=\{\theta_{1},\;\theta_{2},\;\theta_{3}\}$ is then used as the geometric ground-truth for our model.

### 2.2. Training and Test Sets

In this work, we established our dataset based on the RNA 3D Hub non-redundant list (Release 4.21, 7 January 2026), available at https://rna.bgsu.edu/rna3dhub/nrlist/release/rna/4.21/ (accessed on 3 February 2026). First, we retrieved 3120 representative RNA structures with a resolution within 4.0 Å from this list. Next, we employed DSSR (version 2.3.2) [49] to parse these structures and screen for chains that contain experimentally resolved three-way junctions, do not contain junctions with more than three branches, and are shorter than 500 nucleotides. These filtering steps narrowed the initial pool to 625 candidate RNA chains. Subsequently, these candidate chains were clustered using CD-HIT (version 4.6.8) [50] with a sequence identity threshold of 90% to reduce sequence redundancy. This clustering step yielded a final dataset of 139 non-redundant RNA chains. Finally, the curated dataset was randomly partitioned into a training set (111 chains) and an internal test set (28 chains) at an 80:20 ratio, which were used for model optimization and performance evaluation, respectively. The complete lists of PDB IDs and chain identifiers for the training and internal test sets are provided in Supplementary Table S1 and Supplementary Table S2, respectively. It is worth noting that if a chain contains more than one 3WJ, these 3WJs are treated as independent samples for training or test. To characterize the geometric diversity of the dataset and rule out potential biases toward specific structural motifs, we analyzed the statistical distribution of the experimentally determined inter-branch angles (Supplementary Table S3 and Figure S2). The data reveal a broad and continuous conformational landscape for 3WJs, albeit with varying degrees of structural flexibility among the three angles, as evidenced by their distinct standard deviations ($\theta_{1}$ = 23.87°, $\theta_{2}$ = 19.05°, $\theta_{3}$ = 21.85°).

### 2.3. Input Features

The model takes the primary sequence and the secondary structure (in dot-bracket notation) of an RNA that contains only three-way junctions as inputs. To characterize the structure at two hierarchical levels, we construct two complementary graph representations: a fine-grained nucleotide graph embedding residue-level features, and a coarse-grained tree graph embedding topology-level features.

#### 2.3.1. Nucleotide-Level Features

In the fine-grained nucleotide graph, each nucleotide is represented as a node, with edges connecting nucleotides that are either sequentially adjacent or engaged in base-pairing interactions.

Node features. Each node is initialized with a feature vector that concatenates the one-hot encoding of its nucleobase identity (A, U, G, C) and its structural motif label. The motif—categorized as stem (S), hairpin loop (H), bulge loop (B), internal loop (I), multi-branch loop (M), external loop (E), or unclassified single strand (X)—captures the local structural context and serves as a bridge linking to the coarse-grained topological features of the tree graph.

Edge features. We define three edge types for the nucleotide graph, each edge assigned a type-specific feature via one-hot encoding, including backbone edges linking sequentially adjacent nucleotides, nested base-pairing edges (including canonical and non-canonical base pairs), and pseudoknot edges capturing non-nested long-range interactions. While our model focuses on 3WJ inter-stem angles, pseudoknot-like interactions (e.g., kissing loops) can still significantly influence this local geometry. Because coarse-grained tree graphs cannot inherently represent such crossing topologies [45], we explicitly encode pseudoknots as distinct edge channels within the fine-grained nucleotide graph. Although these edges are currently inactive due to our pseudoknot-free dataset (Section 2.1), retaining this architectural interface ensures topological completeness, equipping the framework to achieve the best prediction effect when extended to more complex RNAs.

#### 2.3.2. Topology-Level Features

The tree graph captures the global topological information of the RNA junction as formalized in Section 2.1, where loop regions and helical stems are modeled as nodes and edges, respectively.

Node features. In the tree graph, nodes correspond to RNA loop motifs, and each is encoded by a feature vector consisting of loop segment length- and sequence-derived features (as defined in Table 1), partially adopted from previous tree-graph methodologies [44,47,51]. Here, *M* denotes the number of loop segments in a loop motif. Given that RNA loop motifs include the hairpin loop (M=1), internal loop (M=2), bulge loop (M=1), external loop (M=1 or M=2), and three-way junction loop (M=3), we set the maximum segment capacity in the feature space to M=3. Meanwhile, |$L_{i}$| represents the length of the *i*-th loop segment (1≤i≤M), requiring padding for motifs with *M* < 3 to ensure uniform feature dimensions. Specifically, the loop segment length-derived features (see Table 1 for details) are as follows: the length of each loop segment (|$L_{i}$|), the minimum value of pairwise loop segment lengths (*Min* (|$L_{i}$|, |$L_{j}$|)), sorted loop segment lengths (|${L_{1}}^{\prime }$|, …, |${L_{M}}^{\prime }$|), pairwise loop segment length differences (| |$L_{i}$| − |$L_{j}$| |), length ratios ($\frac{\left|L_{i}\right|}{\left|L_{j}\right|}$), and normalized loop fraction relative to the total loop length ($\frac{\left|L_{i}\right|}{\left|L\right|}$, where |L| = $\sum_{i=1}^{M}L_{i}$, i.e., |L| is the total length of all loop segments in the motif). Complementary to this, the sequence-derived features capture the flexibility of loop segments, specifically the maximum number of consecutive adenine residues (A($L_{i}$)) and the maximum number of consecutive adenine/uracil residues (AU($L_{i}$)) in each loop segment. Subsequently, both the length-derived and sequence-derived features are converted into fixed-length vectors via the Pad (·) operation, which pads all relevant vectors (including those for motifs with *M* < 3) to length 3 with 0 for missing entries to ensure dimension consistency.

Edge features. Each edge in the tree graph represents a helical stem connecting RNA loop regions, with its characteristics captured by a set of edge features summarized in Table 2. These features include the stem length ($\left|L_{stem}\right|$), a geometric property defined as the total number of base pairs in the helical stem; and base-pair composition fractions, a compositional property encompassing the GC pairing fraction ($f_{GC}$), AU pairing fraction ($f_{AU}$), and the non-canonical GU wobble pairing fraction ($f_{GU}$), each normalized relative to the total number of base pairs in the helical stem.

### 2.4. Neural Network Architecture

**Figure 2:**
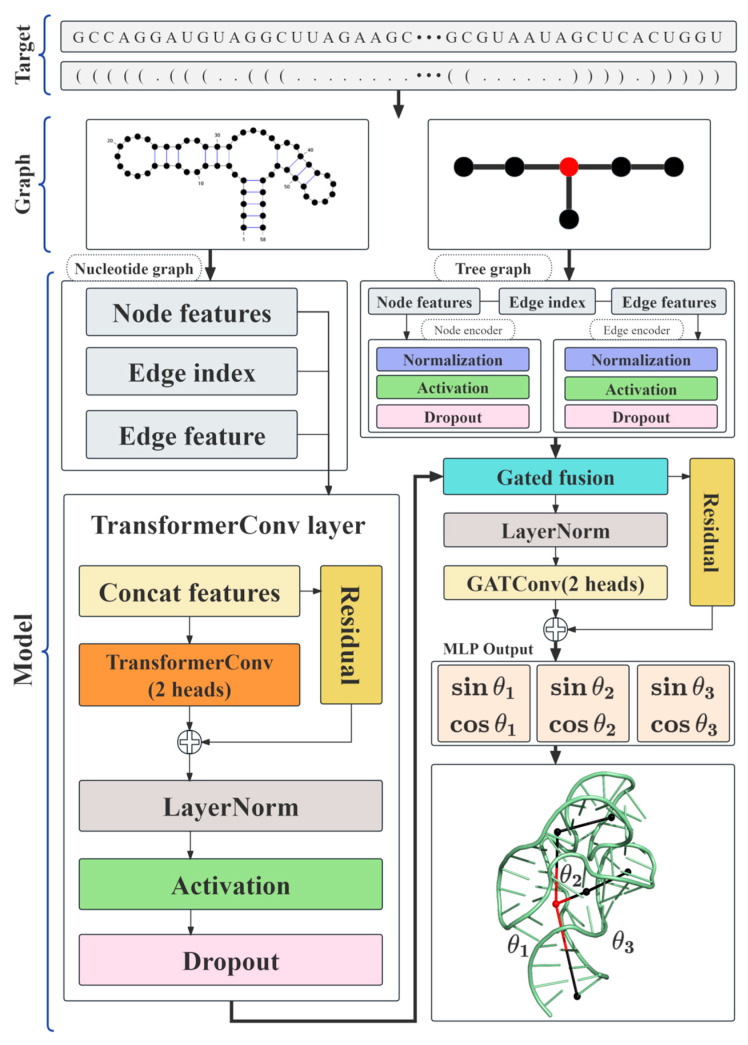
R3J-AGNN architecture overview. Taking RNA primary sequence and secondary structure as input, the model constructs two parallel graphs: a fine-grained nucleotide graph processed via TransformerConv to generate residue embeddings, and a coarse-grained tree graph encoded via node/edge encoders. After gated fusion of these two graph features, the representations are refined by a 2-head GATConv, and an MLP outputs the sine/cosine values of 3WJ inter-branch angles (*θ*_1_/*θ*_2_/*θ*_3_) to support 3D structure reconstruction.

The R3J-AGNN adopts a dual-resolution graph architecture to integrate fine-grained residue-level features with coarse-grained tree-graph-level features. As illustrated in Figure 2, the pipeline consists of three cascaded modules: a nucleotide graph encoding module, a tree graph encoding module, and a cross-scale gated fusion module.

#### 2.4.1. Nucleotide-Graph Encoding Module

This module encodes residue-level sequence and structural information. First, node features are initialized by concatenating sequence base types (A, U, C, G) and structural labels (e.g., hairpin loop types; see Section 2.3.1). These initialized features are then fed into a TransformerConv layer [52] equipped with two attention heads. This message-passing layer aggregates information from neighboring nodes connected by backbone, base-pairing, and pseudoknot edges, yielding enriched residue-level embeddings. This process is illustrated in the left branch of Figure 2.

#### 2.4.2. Tree-Graph Encoding Module

Parallel to the nucleotide graph encoder, this module processes the global topological framework using the tree graph, taking as input the node (Table 1) and edge features (Table 2) defined in Section 2.3.2. These features are projected into a high-dimensional latent space via independent encoders (as shown in the right branch of Figure 2). This projection enables numerical alignment between coarse-grained topological features and fine-grained nucleotide embeddings, laying the groundwork for subsequent feature fusion.

#### 2.4.3. Cross-Scale Gated Fusion

To integrate information across resolutions, we employ an adaptive gated fusion mechanism to combine the encoded residue features ($h_{nucleotide\;}$) from Section 2.4.1 with the encoded tree features ($h_{tree}$) from Section 2.4.2. A learnable gating coefficient dynamically modulates the weight of these dual-resolution features:

(1) $$h=z\cdot h_{nucleotide\;}+(1\;-\;z)\cdot h_{tree}$$

where z∈[0,1] serves as the gating coefficient. The fused feature *h* is then processed via Layer Normalization and a two-head Graph Attention Network [53], followed by a residual connection to generate the final junction representation, as illustrated in Figure 2.

#### 2.4.4. Angle Prediction and Loss Function

The output module is tasked with regressing the three inter-branch angles ($\theta_{1},\theta_{2},\theta_{3}$) of the 3WJ. To address the periodicity of angular values and improve prediction accuracy, we adopt a geometric parameterization strategy [54] instead of directly predicting the angles. Specifically, the MLP output head predicts a 6-dimensional vector that encodes the sine and cosine components of each angle (one pair of sin/cos for each angle), as defined below:

(2) $$\hat{Y}=[sin\theta_{1},\;cos\theta_{1},\;sin\theta_{2},\;cos\theta_{2},\;sin\theta_{3},\;cos\theta_{3}]$$

The model is optimized by minimizing the Mean Squared Error (MSE) loss between the predicted components and their ground-truth counterparts, and the loss calculation process is formalized as follows. Let $\Theta =\{\theta_{1},\;\theta_{2},\;\theta_{3}\}$ denote the set of ground-truth inter-branch angles for valid samples. Consistent with the above parameterization, each ground-truth angle $\theta_{i}$ is mapped to a unit-circle coordinate vector $y_{i}=[sin\theta_{i}\;,\;cos\theta_{i}]$. Let $\hat{y_{i}}=[sin\hat{\theta_{i}}\;,\;cos\hat{\theta_{i}}]$ represent the model’s predicted coordinate vector for $\theta_{i}$. The regression loss (*L*) is then formulated as:

(3) $$L=\frac{1}{N}\sum_{n=1}^{N}\sum_{i=1}^{3}{\left||{\hat{y}}_{n,i}\;-\;y_{n,i}|\right|}_{2}^{2}$$

Here, *N* denotes the number of valid samples in the current batch after masking, and *i* indexes the three inter-branch angles (*i* = 1, 2, 3).

#### 2.4.5. Implementation Details

The dual-resolution architecture is operationalized by deploying multi-head attention-based graph convolutional operators for the fine-grained nucleotide encoding, and standard Graph Attention Network (GAT) formalisms for the coarse-grained topological processing. To ensure seamless cross-scale integration, a uniform hidden representation space is maintained throughout the network hierarchy. Given the restricted scale of the available structural dataset, stochastic regularization via dropout was universally incorporated across all hidden layers to mitigate the risk of overfitting. The network parameters were optimized utilizing the AdamW algorithm (Adam with decoupled weight decay), coupled with an adaptive learning rate decay mechanism conditioned on the convergence plateau of the validation loss. For full reproducibility, a comprehensive specification of the network architecture, optimal hyperparameters, and training protocols is documented in Supplementary Table S4.

### 2.5. Evaluation Metrics

The predicted sine and cosine components are converted back into angular values $\hat{\theta }$ using the two-argument arctangent function atan2. Two metrics are employed to quantify the performance of R3J-AGNN.

#### 2.5.1. Angle Prediction Accuracy (ACC)

We use the angle prediction accuracy (ACC) to quantify model performance under specific tolerance thresholds. Given a tolerance threshold τ (evaluated at 10°, 15°, and 20°), the accuracy is calculated as the proportion of samples that meet the tolerance criterion:

(4) $$ACC(\tau )=\frac{1}{3N}\sum_{i=1}^{N}\sum_{k=1}^{3}I(|{\hat{\theta }}_{i,k}\;-\;\theta_{i,k}|\leq \tau )$$

where I(·) denotes the indicator function. ${\hat{\theta }}_{i,k}$ represents the predicted inter-branch angle for the *k*-th angle of the *i*-th sample, and $\theta_{i,k}$ denotes the corresponding native angle. This metric reveals the model’s robustness across different tolerance thresholds.

#### 2.5.2. Skeleton Root Mean Square Deviation (RMSD)

This metric models the three-way junction (3WJ) as a rigid star topology, consisting of one central junction loop node and three non-junction loop nodes—each of which is individually linked to the central junction loop node via a helical stem. The calculation of this metric involves two straightforward steps: first, geometric reconstruction is performed using native stem lengths and the model’s predicted inter-branch angles; second, the RMSD between the reconstructed 3WJ region and the corresponding region in the native structure is computed, with results reported in angstroms (Å).

## 3. Results

### 3.1. Performance on the Test Set

R3J-AGNN was optimized using 5-fold cross-validation on the training set (Supplementary Figure S3), with hyperparameters selected based on validation performance. As shown in Supplementary Table S5, the model exhibited stable performance across folds, achieving mean accuracies of 0.661, 0.569, and 0.403 under tolerance thresholds of τ = 20°, 15°, and 10°, respectively, which indicates consistent generalization without evident overfitting. The final model was then evaluated on an independent test set containing 28 RNA chains (33 three-way junction instances), where coarse-grained 3D tree graphs were reconstructed using the predicted inter-branch angles.

#### 3.1.1. Accuracy of Angle Prediction

**Figure 3:**
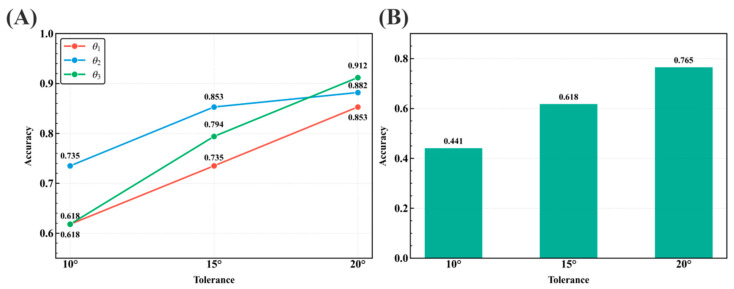
Accuracy of inter-branch angles predicted by R3J-AGNN in the test set. A prediction is considered correct if the absolute angular deviation from the native structure (|∆*θ*|) is less than or equal to the specified tolerance threshold (*τ*). (**A**) Per-angle accuracy. The line plot shows the prediction accuracy trends for each individual angle ($\theta_{1}$, $\theta_{2}$, $\theta_{3}$) across three tolerance thresholds (10°, 15°, and 20°). Specific accuracy values are labeled at each data point. (**B**) Junction-level joint accuracy. The bar plot reports a stricter metric where a junction sample is deemed successfully predicted only if all three angles simultaneously satisfy the tolerance condition.

As shown in Figure 3A, the prediction accuracy for each inter-branch angle ($\theta_{1}$, $\theta_{2}$, $\theta_{3}$) improved with the relaxation of the tolerance threshold *τ*. Specifically, even under the strictest threshold (*τ* = 10°), the model maintained reliable performance, achieving accuracies of 0.618, 0.735, and 0.618 for $\theta_{1}$, $\theta_{2}$, and $\theta_{3}$, respectively. With the threshold relaxed to *τ* = 15°, these corresponding values increased to 0.735, 0.853, and 0.794. At *τ* = 20°, all angle accuracies exceeded 0.85, reaching 0.853 ($\theta_{1}$), 0.882 ($\theta_{2}$), and 0.912 ($\theta_{3}$). These results demonstrate that the model retains robust predictive accuracy above 0.6 for all three inter-branch angles even at *τ* = 10°.

Beyond individual angles, the coherent prediction of all three angles is more critical for revealing the overall 3D conformation of the RNA 3WJ region. Thus, we evaluated junction-level performance using the joint accuracy metric, where a prediction is considered correct only if all three angles simultaneously fall within the specified tolerance. As shown in Figure 3B, the model achieved a joint prediction accuracy of 0.441 even at *τ* = 10°. This accuracy rose to 0.618 at *τ* = 15° and reached 0.765 at *τ* = 20°. Specifically, a joint accuracy of 0.765 indicates that, for 76.5% of test samples, R3J-AGNN achieves a globally consistent orientation of all helical arms with a deviation of ≤20°, yielding a reliable 3D scaffold for downstream geometric reconstruction.

#### 3.1.2. Fidelity of Geometric Reconstruction

**Figure 4:**
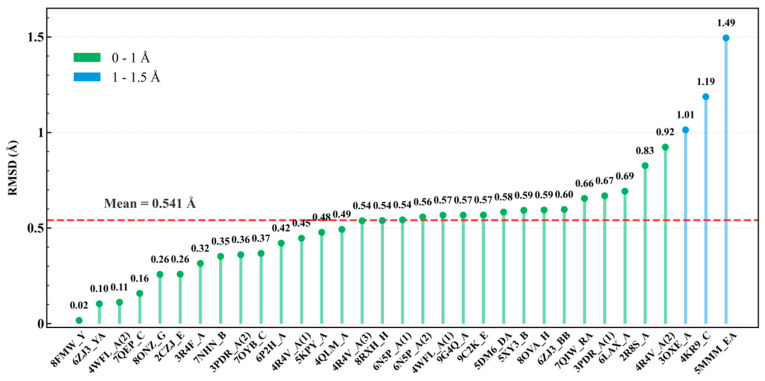
Skeleton RMSD between coarse-grained models constructed from predicted inter-branch angles and corresponding native-abstracted models of the 3WJ regions in the test set. The plot illustrates the geometric deviation between the theoretical topology—reconstructed solely from R3J-AGNN predictions—and the ground truth. Each vertical stem corresponds to a sample labeled by PDB ID; numerical suffixes in parentheses (e.g., (1), (2)) distinguish multiple junctions within the same RNA chain. Bars are color-coded by RMSD range: green (0–1 Å) and blue (1–2 Å). The red dashed line indicates the overall mean RMSD (0.541 Å). This metric serves as a direct proxy for the spatial fidelity of the predicted angular configurations.

To further assess the performance of R3J-AGNN for predicting inter-branch angles, we reconstructed a coarse-grained 3D tree graph for each junction region using the predicted angles and computed the skeleton RMSD against the corresponding experimental structures. As shown in Figure 4, which summarizes the RMSD values across the 33 three-way junction instances from the internal test set, 90.9% of cases (30 out of 33) lie below 1.0 Å (green bars). The remaining three instances fall between 1.0 Å and 1.5 Å (blue bars), with a maximum deviation of 1.49 Å (PDB ID: 5MMM_EA). Notably, all instances have an RMSD below 1.5 Å, yielding an overall mean of 0.541 Å. These results demonstrate that the predicted 3D skeletons are in good agreement with the native structures.

#### 3.1.3. Comparative Case Studies of 3D Skeleton Predictions

**Figure 5:**
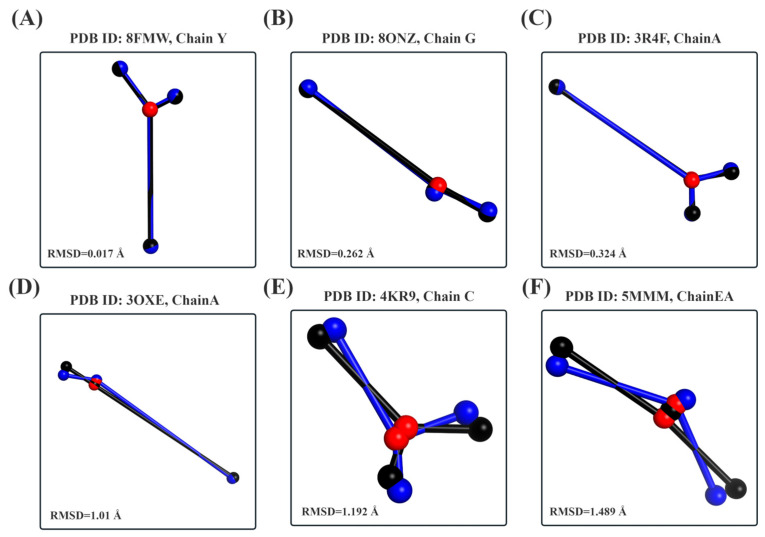
Superimposition of predicted and experimentally determined 3D skeletons for selected 3WJ instances. Native structures are shown in black, R3J-AGNN predictions in blue, and the topological centers of the junctions in red. The top row ((**A**): 8FMW_Y, (**B**): 8ONZ_G, (**C**): 3R4F_A) illustrates high-accuracy predictions with RMSD < 0.35 Å. The bottom row ((**D**): 3OXE_A, (**E**): 4KR9_C, (**F**): 5MMM_EA) displays outlier cases with RMSD > 1.0 Å, highlighting architectural discrepancies.

To further evaluate the geometric fidelity of R3J-AGNN, we superimposed the predicted 3D skeletons onto their native experimental counterparts for selected representative cases (Figure 5). High-performing instances (e.g., 8FMW_Y, 8ONZ_G, and 3R4F_A) exhibited excellent structural congruence with native conformations (RMSD < 0.35 Å), demonstrating the model’s precision in capturing both Y-shaped and linear T-shaped geometries. Conversely, we analyzed three outlier cases (3OXE_A, 4KR9_C, and 5MMM_EA) that exhibited prediction deviations with RMSD values exceeding 1.0 Å. To explore the structural causes of these discrepancies, we examined their secondary topologies and full-length tertiary contexts (Supplementary Figure S4). These challenging instances are characterized by atypical junction topologies, such as inherent instability of single-base-pair stems coupled with a lack of stabilizing long-range tertiary constraints (e.g., 3OXE_A and 4KR9_C), and the pronounced flexibility of long junction loops that harbor complex intra-loop tertiary interactions, including non-canonical base pairing and base stacking (e.g., a 12-nt junction loop in 5MMM_EA).

### 3.2. Comparison with State-of-the-Art RNA 3D Structure Predictors

To evaluate the accuracy of R3J-AGNN, we compared it with four widely used RNA structure prediction tools: trRosettaRNA [36], 3dRNA [55,56], RNAComposer [57], and AlphaFold3 [58]. For each target in our test set, trRosettaRNA, 3dRNA, and RNAComposer were provided with both the primary sequence and the DSSR-derived secondary structure [47], while AlphaFold3 was given only the sequence. For tools that generate multiple candidate models (AlphaFold3, 3dRNA, and trRosettaRNA), we selected the top-ranked model according to their internal scoring functions; for RNAComposer, which outputs a single structure, the sole model was used directly.

To ensure the validity of geometric comparisons, only predictions in which the secondary structure of the three-way junction region exactly matched the experimental structure were retained. The necessity of this strict filtering is highlighted by the performance of AlphaFold3. Out of the 33 RNA 3WJ instances evaluated in our independent test set, AlphaFold3 predicted the correct secondary topology for only eight instances. As illustrated in Supplementary Figure S5, AlphaFold3 achieved remarkably low global RMSD values (mean of 5.532 Å) for these eight topologically correct targets. In stark contrast, its 3D predictions for the remaining instances with incorrect secondary structures exhibited massive global RMSDs (e.g., erroneously folding 3WJ branches into simple hairpins). Consequently, to ensure a meaningful geometric evaluation, for each of these eight structurally valid targets, the best-performing competitor model was selected and benchmarked directly against R3J-AGNN.

As summarized in Table 3, R3J-AGNN achieved the lowest mean absolute error (MAE) across the three inter-branch angles in five out of the eight evaluated targets. For example, on target 3PDR, R3J-AGNN yielded an MAE of 4.56°, substantially outperforming RNAComposer (7.84°). On target 4QLM, it attained an MAE of only 3.32°, compared to 9.17° for AlphaFold3. For target 4R4V, R3J-AGNN achieved an MAE of 1.22°, outperforming RNAComposer’s 3.11° error. On target 7QEP, R3J-AGNN maintained a remarkably low MAE of 1.18°, whereas RNAComposer exhibited a large MAE of 9.98°. Even in cases where trRosettaRNA achieved sub-degree precision (e.g., 0.93° on 4WFL and 1.42° on 6P2H), R3J-AGNN remained competitive, with reasonable MAEs of 3.75° and 2.07° on the same targets, respectively. Detailed results for all four predictors are provided in Supplementary Figure S6.

### 3.3. Ablation Study: Architectural and Feature Contributions

To systematically evaluate the contribution of each architectural component and feature design, we conducted an ablation study on the internal test set. The full R3J-AGNN model integrates two attention-based graph neural networks operating at different structural resolutions, a fine-grained nucleotide graph and a coarse-grained tree graph, whose embeddings are combined through a gated fusion mechanism. We constructed several ablation variants targeting the key architectural and feature design choices of R3J-AGNN. To evaluate the necessity of multi-scale structural modeling, two single-resolution variants were introduced: R3J-AGNN (-Nucleotide-graph), in which the fine-grained nucleotide graph branch was removed, and R3J-AGNN (-Tree-graph), where the coarse-grained tree graph branch was excluded. To assess the role of attention-based message passing, we designed R3J-AGNN (-Transformer), in which all attention-based graph convolution layers were replaced with standard GCN operators. To examine the effectiveness of adaptive cross-resolution integration, the gated fusion module was replaced with direct embedding concatenation, yielding R3J-AGNN (-Gated fusion). Finally, to address potential redundancy in tree-graph node features, we constructed a feature ablation variant, R3J-AGNN (len-only), which retains only the raw junction loop-length features while removing derived features such as length ratios and pairwise differences. All ablated models were trained using the same training protocol and hyperparameter settings as the full R3J-AGNN model unless otherwise specified. Model performance was evaluated using the joint angle accuracy under tolerance thresholds of 10°, 15°, and 20°.

As summarized in Table 4, ablation analyses consistently demonstrate the importance of hierarchical structural representations and feature integration in R3J-AGNN. Among the single-resolution baselines, the nucleotide-only model (R3J-AGNN (-Tree-graph)) showed limited predictive capability, achieving accuracies of 0.206 and 0.588 at the 10° and 20° thresholds, respectively. In contrast, the tree-only model (R3J-AGNN (-Nucleotide-graph)) substantially improved performance (0.382 at 10° and 0.677 at 20°), highlighting the dominant role of coarse-grained topological constraints in defining RNA junction geometry. The architectural ablations further highlight the importance of the proposed information integration mechanisms. Eliminating the attention module (R3J-AGNN (-Transformer)) produced the lowest performance among architectural variants, suggesting reduced capability in modeling long-range dependencies. Similarly, removing the gating mechanism (R3J-AGNN (-Gated fusion)) caused a substantial accuracy drop at the strictest threshold (0.265 at 10°), indicating that adaptive feature fusion is essential for preserving fine-grained geometric information. Feature ablation using only length-related descriptors (R3J-AGNN (len-only)) also reduced performance across all thresholds (0.630, 0.529, and 0.294). This decline aligns with the permutation importance analysis of node features (Supplementary Figure S7), which shows that length-based descriptors contribute least to prediction accuracy among all feature groups. Together, these findings demonstrate that geometric prediction cannot be explained solely by junction size and requires richer structural representations. Notably, the full R3J-AGNN model consistently achieved the best performance across all evaluation criteria, reaching accuracies of 0.765, 0.618, and 0.441 at 20°, 15°, and 10°, respectively. Compared with the strongest single-resolution variant (R3J-AGNN (-Nucleotide-graph)), the full model achieved an absolute improvement of 8.8% at τ = 20° and maintained a clear advantage under the strictest tolerance (0.441 vs. 0.382). Collectively, these results demonstrate that accurate RNA junction geometry prediction emerges from the synergistic integration of hierarchical structural representations and adaptive cross-resolution feature fusion.

## 4. Discussion

In this study, we propose R3J-AGNN, a dual-resolution graph neural network designed to predict inter-branch angles in RNA three-way junctions. Evaluation on an independent test set of 33 3WJ instances confirmed the model’s good geometric fidelity: 76.5% joint prediction accuracy (defined as all three inter-branch angles falling within a 20° tolerance) and a mean skeleton RMSD of 0.541 Å, with 90.9% of instances exhibiting deviations below 1.0 Å, confirming its ability to reproduce native junction geometry.

Analysis of the prediction patterns reveals two notable geometric trends. First, the model achieves the highest accuracy for $\theta_{2}.$ Statistical analysis of the dataset (Supplementary Table S3) indicates that $\theta_{2}$ exhibits the lowest structural variance (standard deviation: 19.05°, compared to 23.87° for $\theta_{1}$ and 21.85° for $\theta_{3}).$ From a structural perspective, this suggests that the helical stems defining $\theta_{2}$ are subject to stronger geometric constraints and possess a more restricted conformational space, which inherently simplifies the predictive learning task. Second, pairwise error analysis (Supplementary Figure S8) reveals significant positive correlations between $\theta_{1}$/$\theta_{2}$ (*r* = 0.417, *p* = 0.016) and $\theta_{2}$/$\theta_{3}$ (*r* = 0.449, *p* = 0.009). The relatively weaker correlation between $\theta_{3}$/$\theta_{1}$ (*r* = 0.332, *p* = 0.059) implies that $\theta_{2}$ serves as a structural pivot during error propagation. This coordinated pattern reflects the topological closure constraint of three-way junctions, where deviations must collectively satisfy helical stem compatibility. Importantly, while this general geometric coupling holds true, the moderate correlation coefficients (*r* < 0.5) indicate that the linkage is not perfectly rigid. As evidenced by the scatter plots (Figure S6), instances do exist where one specific angle is poorly predicted despite the others remaining highly accurate. These localized deviations typically arise from branch-specific structural idiosyncrasies, such as exceptional flexibility or uncharacterized non-canonical interactions within a single junction loop, which perturb the local geometry without completely disrupting the global topological constraint of the other two stems.

Structurally, these rigid geometric constraints are heavily governed by a hierarchy of higher-order interactions [59,60]. As explicitly visualized in Supplementary Figure S9, we observe that extreme inter-branch angles, particularly those approaching 180°, which are characteristic of nearly linear alignments, are frequently driven by coaxial stacking between adjacent branches. This continuous stacking is mechanically stabilized at the local level by junction-loop base triples (e.g., minor-groove interactions) and further locked by long-range tertiary contacts, such as kissing loop interactions [59,60]. Together, these local and long-range physical anchors establish the strict geometrical boundaries that our model implicitly learns to predict from structural context.

Benchmarking against four state-of-the-art tools (trRosettaRNA, 3dRNA, RNAComposer, and AlphaFold3) across eight diverse 3WJ targets revealed R3J-AGNN’s specialization. It outperformed competitors with lower mean absolute error (MAE) on five targets. For instance, on target 4QLM, R3J-AGNN achieved an MAE of 3.32° versus AlphaFold3’s 9.17°; on 7QEP, R3J-AGNN reported an MAE of 1.18° versus RNAComposer’s 9.98°. While trRosettaRNA excelled on target 4WFL with an MAE of 0.93°, R3J-AGNN maintained robust performance at 3.75°, underscoring consistent accuracy across diverse 3WJ instances. This contrasts with conventional physics-based methods, which often yield topologically inconsistent junctions due to rugged energy landscapes, and general deep learning frameworks, which lack explicit optimization for 3WJ-specific geometry.

Furthermore, ablation studies quantitatively validated the necessity of dual-resolution integration. At the 20° tolerance threshold, the full model attained 76.5% joint accuracy, outperforming the nucleotide-level (58.8%) and tree-graph-level (67.7%) baselines. Gains widened at the stricter 10° threshold (44.1% vs. 20.6% and 38.2%, respectively), confirming that complementary information from both resolutions is indispensable for high-fidelity 3WJ modeling.

However, there are several limitations. First, the dataset (139 non-redundant 3WJ-containing chains from RNA 3D Hub) for training and testing is relatively small, potentially restricting generalizability to rare junction topologies. Second, the current framework is exclusive to 3WJs and has not been extended to other multi-way junctions (e.g., four-way junctions). Third, current evaluations rely on geometric metrics (angular accuracy and RMSD), and thus future efforts should integrate predicted angles as constraints in full-length RNA folding pipelines to quantify the downstream impact on tertiary structure prediction. Fourth, to accommodate exceptionally short stems and maintain a unified metric across the dataset, stem orientation is currently defined using a single closing base pair. While this captures the immediate local geometry at the junction core, it is inherently susceptible to local structural distortions. As illustrated in Supplementary Figure S1, defining the helical axis with a single base pair yields a wider inter-angle range (36.69°) compared to a three-base-pair fitting (17.33°), which spatially averages local variations to produce more convergent values. Furthermore, different branches exhibit varying sensitivities to the fitting window size: for example, $\theta_{2}$ remains relatively stable (Δ = 3.8°), whereas $\theta_{3}$ shows pronounced sensitivity (15.6°), indicating that local structural distortions are unevenly distributed among the three stems. Therefore, future optimizations could implement adaptive multi-base-pair fitting for longer stems to extract more robust, globally representative orientation vectors. Fifth, the current nucleotide graph primarily encodes canonical secondary base pairings, and future iterations of R3J-AGNN could explicitly incorporate non-canonical interactions (e.g., the branch-loop base triples) as distinct topological edge types. Evolving into a multi-relational graph would allow the model to natively perceive these higher-order stabilizing forces, thereby further improving the accurate prediction of atypical angular variations and rare junction topologies.

## 5. Conclusions

In this work, we presented R3J-AGNN, a dual-resolution graph neural network specifically developed for predicting the inter-branch angles of RNA three-way junctions (3WJs) directly from secondary structure information. By synergistically fusing nucleotide-level interaction details with global topological features through a gated fusion mechanism, the model achieves high-precision angular prediction and reconstructs coarse-grained 3WJ skeletons with exceptional fidelity to native geometries. Rigorous evaluation confirms that this multi-scale integration is essential, with R3J-AGNN significantly outperforming single-resolution baselines and state-of-the-art RNA structure prediction tools across diverse benchmarks. This study provides a targeted and effective methodology for resolving the key geometric features of RNA 3WJs, holding promise for advancing broad research efforts in RNA tertiary structure prediction and functional interpretation.

## Figures and Tables

**Table 1:** Node features utilized in the coarse-grained tree-graph.

Node Feature	Description
${Pad}^{\;a}({\left[|L_{i}|\right]}_{1\;\leq \;i\;\leq \;M^{c}}^{b}$, 3, 0)	Loop length in the *i*-th loop segment, and *i* is labeled according to the 5′ to 3′ direction of the RNA chain.
$Pad{\left([Min(|L_{i}|,|L_{j}|)\right]}_{1\;\leq \;i\;<\;j\;\leq \;M}$, 3, 0)	Minimum value of the lengths of the *i*-th and *j*-th loop segments.
$Pad{\left([|{L_{i}}^{\prime }|\right]}_{1\;\leq \;i\;\leq \;M}$, 3, 0)	Loop lengths sorted in ascending order.
$Pad{\left([||L_{i}|-|L_{j}||\right]}_{1\;\leq \;i\;<\;j\;\leq \;M}$, 3, 0)	Absolute difference in the lengths of *i*-th and *j*-th loop segment.
$Pad{\left([\frac{\left|L_{i}\right|}{\left|L_{j}\right|}\right]}_{1\;\leq \;i\;<\;j\;\leq \;M}$, 3, 0)	Ratio of the length of *i*-th loop segment and *j*-th loop segment.
$Pad{\left([\frac{\left|L_{i}\right|}{\left|L\right|}\right]}_{1\;\leq \;i\;\leq \;M}$, 3, 0)	Normalized fraction of the length of *i*-th loop segment relative to the total length of all loop segments, i.e., $\left|L\right|$=$\sum_{i=1}^{M}\left|L_{i}\right|$.
$Pad{\left([AU(L_{i})\right]}_{1\;\leq \;i\;\leq \;M}$, 3, 0)	Maximum number of consecutive Adenine (A) or Uracil (U) residues in the *i*-th loop segment.
$Pad{\left([A(L_{i})\right]}_{1\;\leq \;i\;\leq \;M}$, 3, 0)	Maximum number of consecutive Adenine (A) residues in the *i*-th loop segment.

^a^ Pad(·) is a standard padding operation that pads all vectors to a fixed length of 3, where 3 matches the number of loop segments in a three-way junction, and fills missing entries with the value 0. ^b^ Square brackets [ ] denotes a feature vector, where each element corresponds to a loop segment or a pairwise combination of loop segments, representing ordered, multi-dimensional features. ^c^ *M* represents the number of loop segments in the RNA motif (e.g., *M* = 1 for hairpin loops, *M* = 2 for internal loops, *M* = 3 for three-way junctions, and *M* = 1 or *M* = 2 for exterior loops).

**Table 2:** Edge features utilized in the coarse-grained tree-graph.

Edge Feature	Description
$\left|L_{stem}\right|$	Stem length, calculated by the total number of base pairs in the helical stem.
$f_{GC}$	GC base pairing fraction, the normalized frequency of Guanine-Cytosine (G-C) base pairs relative to the total base pairs in the helical stem, defined as $\frac{N_{GC}}{\left|L_{stem}\right|}$.
$f_{AU}$	AU base pairing fraction, the normalized frequency of Adenine-Uracil (A-U) base pairs relative to the total base pairs in the helical stem, defined as $\frac{N_{AU}}{\left|L_{stem}\right|}$.
$f_{GU}$	GU base pairing fraction, the normalized frequency of non-canonical Guanine-Uracil (G-U) wobble pairs relative to the total base pairs in the helical stem, defined as $\frac{N_{GU}}{\left|L_{stem}\right|}$.

**Table 3:** Comparison of inter-branch angle predictions by R3J-AGNN against the top result from four RNA 3D structure predictors (i.e., TrRosettaRNA, 3dRNA, RNAComposer and AlphaFold3) for each target. Best results are highlighted in bold.

PDB ID	Method	$\left|{∆\theta }_{1}\right|$ ^a^	$\left|{∆\theta }_{2}\right|$	$\left|∆\theta_{3}\right|$	MAE ^b^
3PDR	R3J-AGNN	**0.48°**	8.19°	**5.42°**	**4.56°**
	RNAComposer	3.12°	**7.78°**	11.75°	7.84°
4QLM	R3J-AGNN	**6.68°**	**0.42°**	**2.87°**	**3.32°**
	AlphaFold3	11.95°	1.81°	13.76°	9.17°
4R4V	R3J-AGNN	**0.19°**	**0.87°**	2.59°	**1.22°**
	RNAComposer	4.66°	2.48°	**2.18°**	3.11°
4WFL	R3J-AGNN	6.04°	2.07°	3.15°	3.75°
	trRosettaRNA	**0.37°**	**1.39°**	**1.02°**	**0.93°**
6P2H	R3J-AGNN	2.42°	**2.00°**	**1.80°**	2.07°
	trRosettaRNA	**0.03°**	2.09°	2.13°	**1.42°**
7QEP	R3J-AGNN	**1.93°**	**0.06°**	**1.55°**	**1.18°**
	RNAComposer	14.97°	6.04°	8.93°	9.98°
8FMW	R3J-AGNN	**3.52°**	**0.77°**	5.26°	**3.18°**
	RNAComposer	5.47°	0.89°	**4.58°**	3.65°
8RXH	R3J-AGNN	0.10°	7.14°	11.00°	6.08°
	trRosettaRNA	**2.71°**	**0.57°**	**3.28°**	**2.19°**

^a^ Absolute deviation between predicted and experimental angles. ^b^ MAE is defined as the average absolute deviation across the three inter-branch angles ($\theta_{1}$, $\theta_{2}$, $\theta_{3}$).

**Table 4:** Ablation analysis of R3J-AGNN on the independent test set. Joint angle prediction accuracy under tolerance thresholds *τ* = 20°, 15°, and 10°. The best performance is shown in bold.

Model	Tolerance Threshold		
	20°	15°	10°
R3J-AGNN (-Tree-graph)	0.588	0.441	0.206
R3J-AGNN (-Nucleotide-graph)	0.677	0.588	0.382
R3J-AGNN (-Transformer)	0.500	0.412	0.324
R3J-AGNN (-Gated fusion)	0.706	0.618	0.265
R3J-AGNN (len-only)	0.630	0.529	0.294
R3J-AGNN	**0.765**	**0.618**	**0.441**

## Data Availability

R3J-AGNN and relevant databases are available at the following website: https://github.com/RNA-folding-lab/R3J-AGNN (accessed on 3 February 2026).

## Supplementary Materials

The following supporting information can be downloaded at: https://www.mdpi.com/article/10.3390/biology15060457/s1, Figure S1: Effect of base-pair window size on stem orientation and inter-branch angle definition; Figure S2: Statistical distribution and predictive performance of inter-branch angles; Figure S3: Training and validation loss curves of the R3J-AGNN model; Figure S4: Secondary structures and tertiary contexts of the selected RNA three-way junctions; Figure S5: Box plots of RMSD distributions for AlphaFold3-predicted RNA structures against their native experimental structures; Figure S6: Geometric accuracy analysis for eight representative targets; Figure S7: Permutation importance analysis of node features in the coarse-grained tree graph; Figure S8: Pairwise correlation analysis of absolute prediction errors for the three inter-branch angles; Figure S9: Visualization of tertiary interactions stabilizing RNA three-way junctions with coaxial-stacking; Table S1: PDB ID, chain and length of RNAs in training set; Table S2: PDB ID, chain and length of RNAs in test set; Table S3: Statistical distribution of the inter-branch angles in the 3WJ dataset; Table S4: Hyperparameters of the R3J-AGNN model; Table S5: Five-fold cross-validation accuracy of R3J-AGNN on validation sets under different angular tolerance thresholds.
