# Supplementary material for: R3J-AGNN: GNN-Based Prediction of Inter-Branch Angles in RNA Three-Way Junctions from Secondary Structure
Source: Biology (Basel). 2026 Mar 11;15(6):457. doi: 10.3390/biology15060457 (PMC13023823; doi:10.3390/biology15060457)
Supplement: Supplementary file 1 [file biology-15-00457-s001.zip › biology-4164559-supplementary.pdf]

Supplementary Material for

R3J-AGNN: GNN-based prediction of inter-branch angles in RNA three-way junction

Hu Yang[1†], Ning Qiao[2†], Bengong Zhang[1], Ya-Zhou Shi[1*], Ya-Lan Tan[1,2*]

[1]Research Center of Nonlinear Science, School of Mathematical and Physical Sciences, Wuhan Textile University, Wuhan 430200, China
[2]School of Biomedical Engineering and Health, Wuhan Textile University, Wuhan 430200, China

---

[†]These authors contributed equally to this work.

*Corresponding authors: yltan@wtu.edu.cn (to YLT) and yzshi@wtu.edu.cn (to YZS).

Table S1 PDB ID, chain and length of RNAs in training set.

PDB ID	Chain	Length	PDB ID	Chain	Length	PDB ID	Chain	Length
1J2B	A	77	5DDP	A	61	7QR4	B	69
1KQS	B	122	5DGE	KA	121	7R81	C	120
1KUQ	A	54	5GAD	D	120	7UY6	G	156
1UN6	D	61	5GM6	D	117	7WAZ	C	115
1Y26	A	71	5IMQ	H	123	7WB1	C	121
1Y27	A	68	5MMM	J	121	7YOJ	D	174
2OIU	A	71	5O61	C	118	8A22	E	136
2V3C	E	96	5T7V	O	114	8A3W	OA	153
3D2V	A	77	5TPY	A	71	8CD1	F	120
3E5C	A	53	5XXB	B	118	8CRX	XA	120
3EGZ	B	65	6J6G	Q	179	8F4O	B	64
3IVN	A	69	6MWN	A	92	8GZP	B	67
3IWN	A	93	6O8W	W	116	8HKU	B	122
3J7P	B	120	6OL3	A	111	8ITS	A	46
3J9W	HA	112	6PRV	A	58	8J7R	B	97
3K0J	E	87	6RM3	B	118	8ONY	G	58
3KTW	A	96	6SKG	BA	125	8OVA	D	119
3LA5	A	71	6UES	A	119	8P7X	E	108
3MOJ	A	69	6XKO	A	95	8PV7	D	119
3NKB	B	64	6XU6	CB	120	8RD8	K	115
3P49	A	169	6XYW	RA	118	8SH5	C	88
3RKF	C	67	6YSI	BA	115	8T29	A	90
3SKI	A	68	6ZDP	B	67	8UMF	B	121
3SUX	A	101	6ZDQ	B	65	8ZAU	A	69
4LVW	A	89	6ZU5	B	119	9AUF	B	146
4O26	C	47	7JIL	Z	110	9AXU	D	119
4P8Z	A	188	7K16	A	51	9BUP	JA	118
4PQV	A	68	7KGA	B	90	9CAI	I	119
4QK9	A	117	7KVT	A	83	9E6Q	B	129
4UYK	C	134	7MQA	C	177	9FN2	A	58
4V8P	JE	120	7OBR	A	249	9FXO	E	121
4W90	B	101	7OLC	C	119	9G4R	A	47
4XNR	A	71	7OYC	D	119	9H91	G	121
4YAZ	A	84	7PKT	WA	184	9I05	IC	178
5AOX	C	85	7PKT	YA	149	9I5V	P	104
5BJO	A	36	7PWG	B	117	9NTA	B	128
5D8H	A	74	7QCA	B	119	9QT5	B	114

Table S2 PDB ID, chain and length of RNAs in test set.

PDB ID	Chain	Length	PDB ID	Chain	Length	PDB ID	Chain	Length
2CZJ	E	62	5KPY	A	71	7QEP	C	119
2R8S	A	159	5MMM	EA	106	7QIW	RA	120
3OXE	A	88	5XY3	B	117	8FMW	Y	112
3PDR	A	161	6LAX	A	55	8ONZ	G	59
3R4F	A	66	6N5P	A	127	8OVA	H	119
4KR9	C	39	6P2H	A	69	8RXH	H	120
4QLM	A	108	6ZJ3	YA	92	9C2K	E	72
4R4V	A	186	6ZJ3	BB	120	9G4Q	A	52
4WFL	A	107	7NHN	B	114			
5DM6	DA	122	7OYB	C	120			

Table S3 Statistical distribution of the inter-branch angles in the 3WJ dataset.

Angle	Mean	Median	Standard Deviation
θ_1	126.91°	124.60°	23.87°
θ_2	113.27°	107.85°	19.05°
θ_3	119.82°	114.98°	21.85°

Table S4 Hyperparameters of the R3J-AGNN model.

Parameter	Value
Number of TransformerConv layers	3
Number of GAT layers	5
Hidden dimension (shared across all graph layers)	200
Dropout rate	0.50
Number of attention heads	2
Optimizer	AdamW
Weight decay	1×10^{-4}
Learning rate	6×10^{-5}
Batch size	1
Training epochs	100

Table S5 Five-fold cross-validation accuracy of R3J-AGNN on validation sets under different angular tolerance thresholds.

**Fold**	**Accuracy**		
	**20°**	**15°**	**10°**
Fold 1	0.735	0.677	0.412
Fold 2	0.629	0.514	0.429
Fold 3	0.677	0.588	0.412
Fold 4	0.552	0.517	0.310
Fold 5	0.710	0.548	0.452
Mean±standard	0.661±0.071	0.569±0.062	0.403±0.050

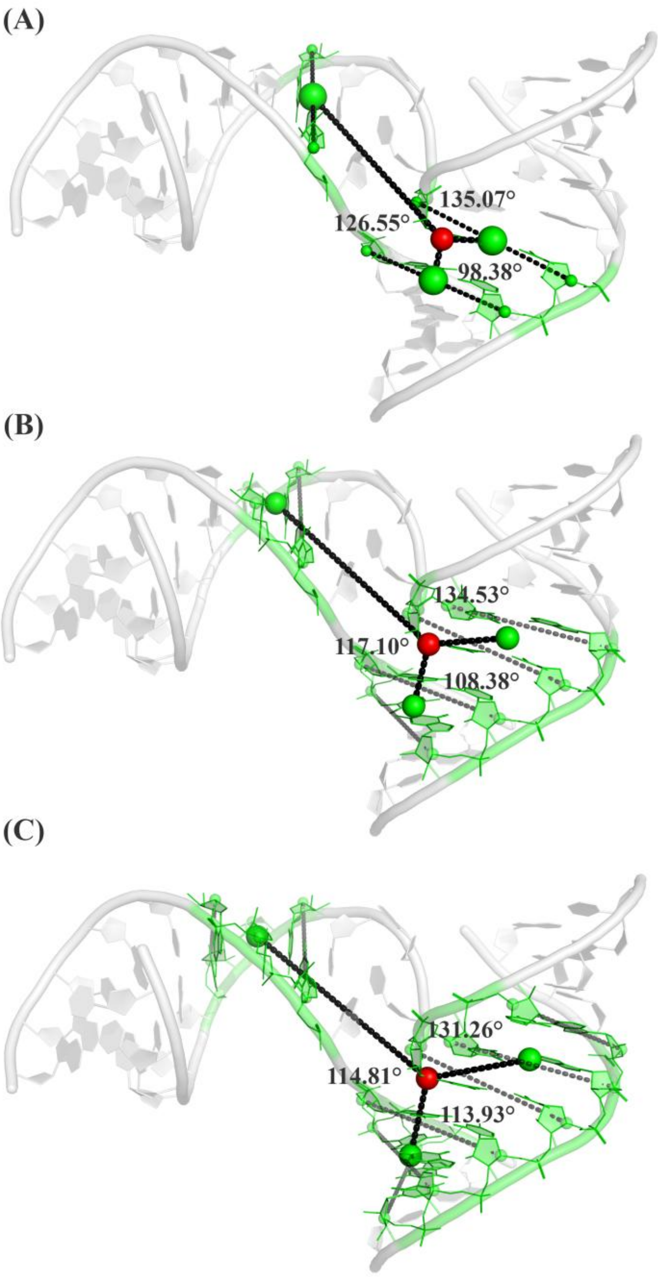

Figure S1. Effect of base-pair window size on stem orientation and inter-branch angle definition. Using a representative RNA three-way junction (PDB ID: 3R4F, Chain A), stem orientation vectors (black dashed lines) and the resulting inter-branch angles are compared when the helical axis is defined by fitting (A) one closing base pair, (B) two consecutive base pairs, and (C) three consecutive base pairs.

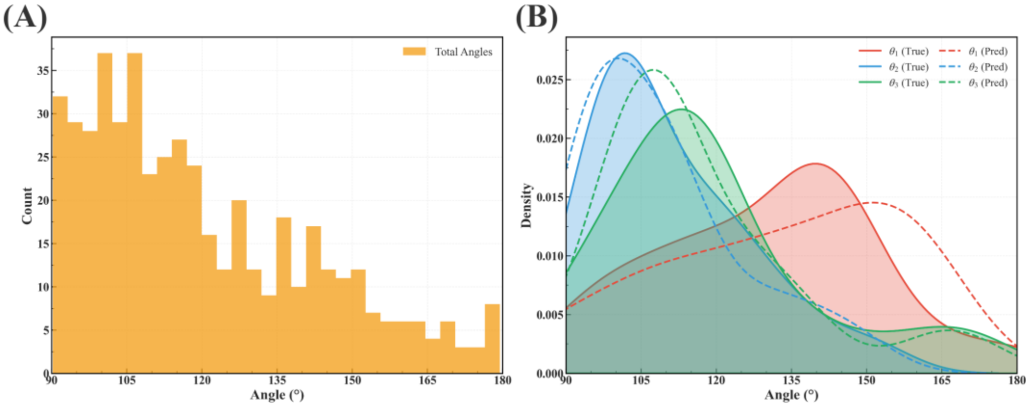

Figure S2. Statistical distribution and predictive performance of inter-branch angles. (A) Global distribution of all experimentally determined inter-branch angles across the entire dataset. (B) Comparison between experimental (solid lines) and R3J-AGNN predicted (dashed lines) angle distributions on the internal test set. Angles θ_1, θ_2, and θ_3 are represented in red, blue, and green, respectively. The predicted angle distributions closely align with the broad experimental profiles without exhibiting significant regression toward a narrow mean. This robust alignment demonstrates the model's capacity to genuinely learn and generalize across diverse junction topologies.

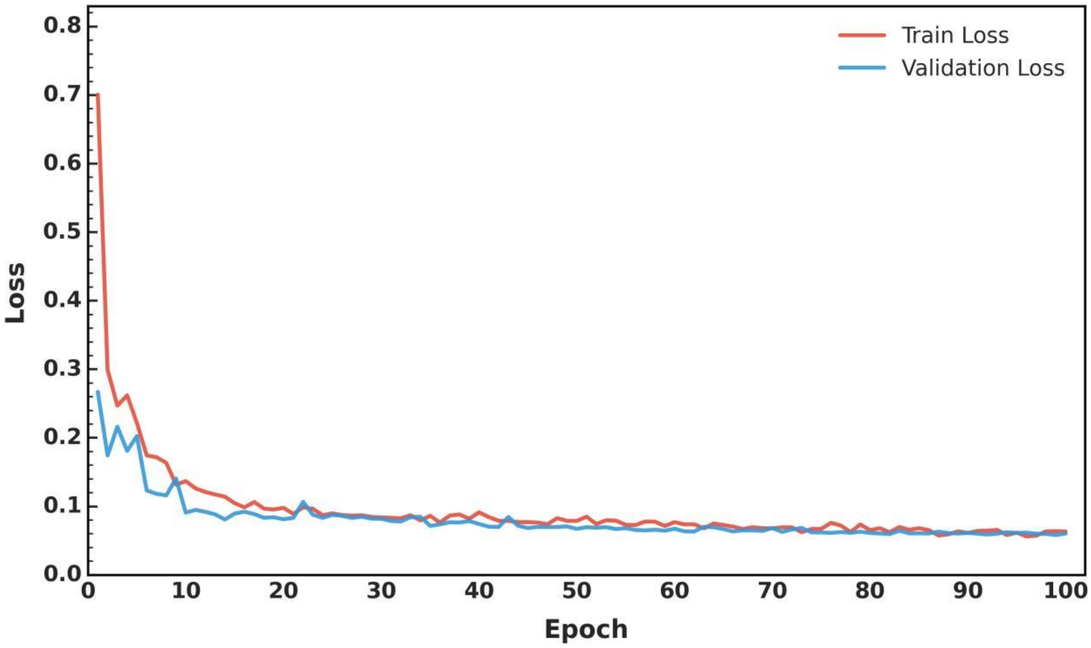

Figure S3. Training and validation loss curves of the R3J-AGNN model. The primary dataset was randomly partitioned into a training set and an independent test set . To optimize the model, a 5-fold cross-validation strategy was employed on the training set. This plot illustrates the Mean Squared Error (MSE) loss curves for the best-performing fold (where N_{train}=88 and N_{val}=23). The red line represents the training loss, while the blue line represents the validation loss during this specific fold.

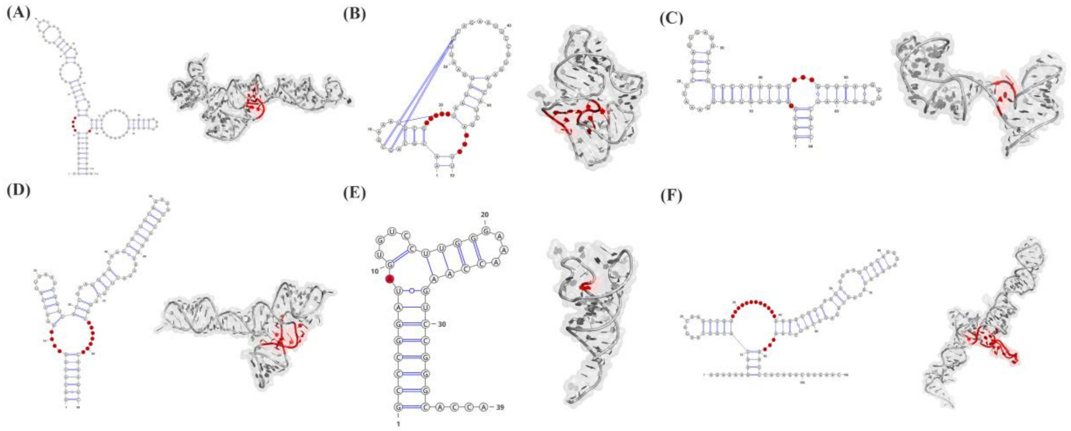

Figure S4. Secondary structures and tertiary contexts of the selected RNA three-way junctions. For each instance, the secondary structure (left) highlights the junction loop nucleotides in red, while the full-length tertiary structure (right) shows the native 3D conformation in gray surface representation with the junction region highlighted in red. The instances correspond to those analyzed in Figure 5: top row (A: 8FMW_Y, B: 8ONZ_G, C: 3R4F_A) represent high-accuracy predictions; bottom row (D: 3OXE_A, E: 4KR9_C, F: 5MMM_EA) represent outlier cases with higher RMSD, characterized by asymmetrical or atypical loop sizes.

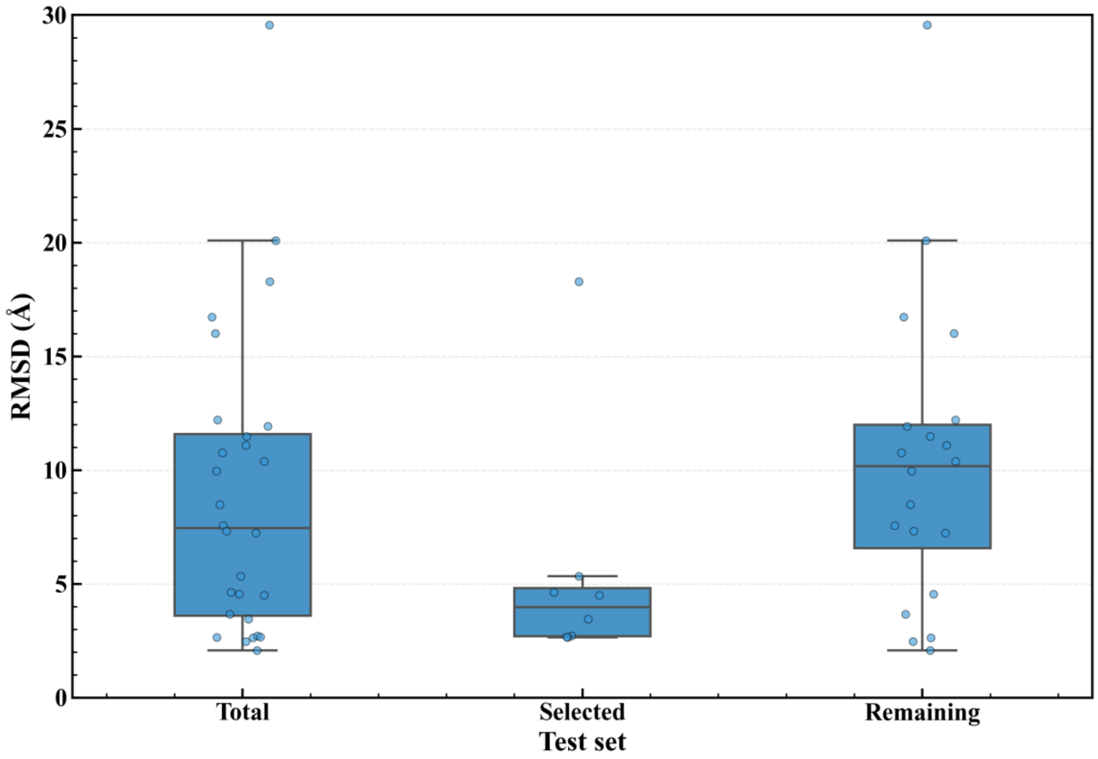

Figure S5. Box plots of RMSD distributions for AlphaFold3-predicted RNA structures against their native experimental structures. The plots display the Raw RMSD (the global RMSD calculated across all aligned atoms without spatial filtering). Performance is evaluated across three sample subsets: the entire independent test set (Total), the eight representative benchmarking targets (Selected), and the remaining structures (Remaining). The generally high raw RMSD values across the subsets indicate that AlphaFold3 struggles to accurately capture the global topologies of these complex RNA structures, likely suffering from severe conformational deviations in flexible regions.

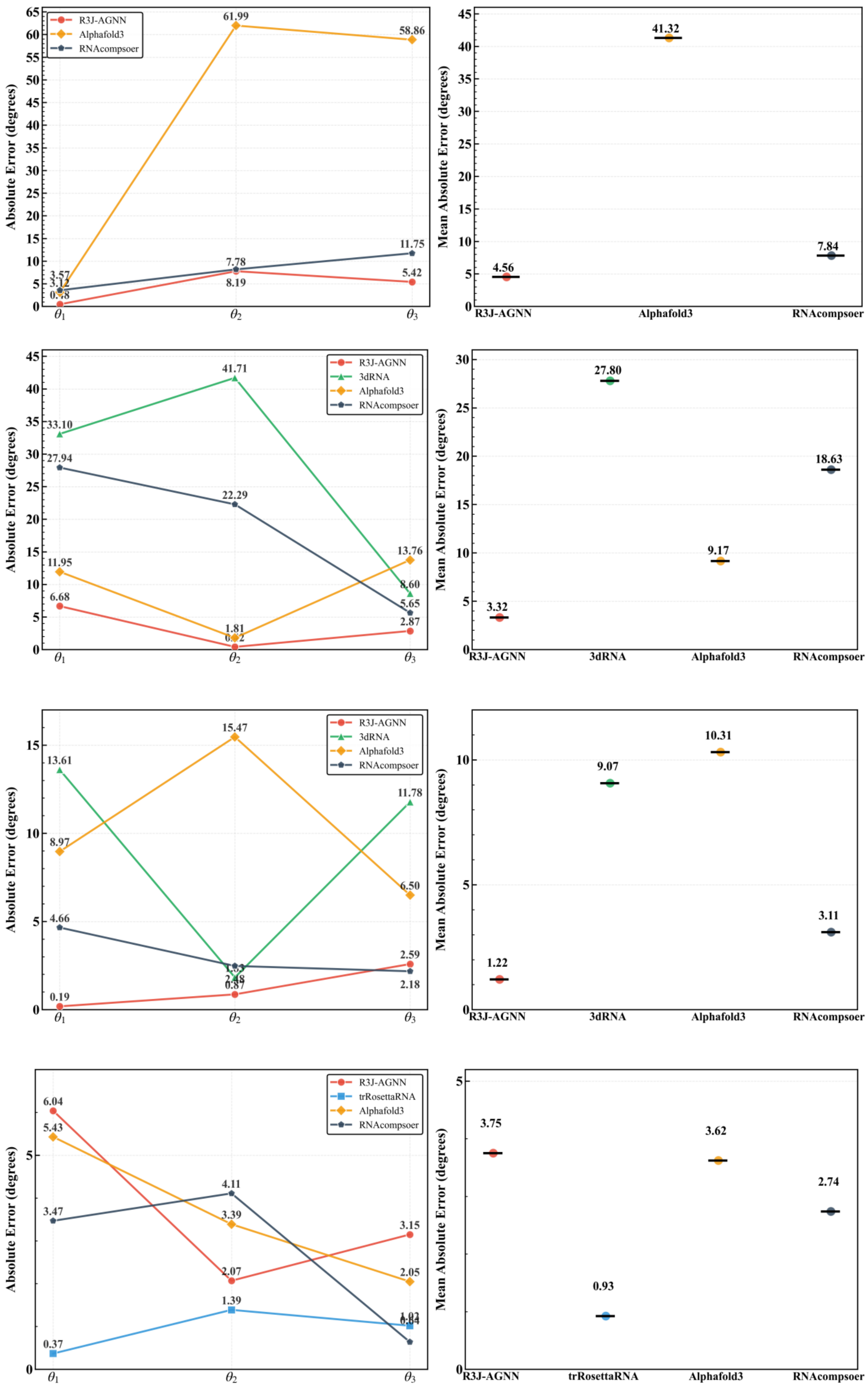

Absolute Error (degrees)
Mean Absolute Error (degrees)
R3J-AGNN
3dRNA
trRosettaRNA
Alphafold3
RNAcompsoer
θ_1
θ_2
θ_3
61.99
58.86
3.57
3.12
7.78
8.19
11.75
5.42
4.56
41.32
7.84
41.71
33.10
27.94
22.29
11.95
6.68
1.81
13.76
8.60
5.65
2.87
3.32
27.80
9.17
18.63
15.47
13.61
8.97
4.66
0.19
11.78
6.50
2.59
2.18
1.22
9.07
10.31
3.11
6.04
5.43
3.47
4.11
3.39
2.07
1.39
0.37
3.15
2.05
3.75
0.93
3.62
2.74

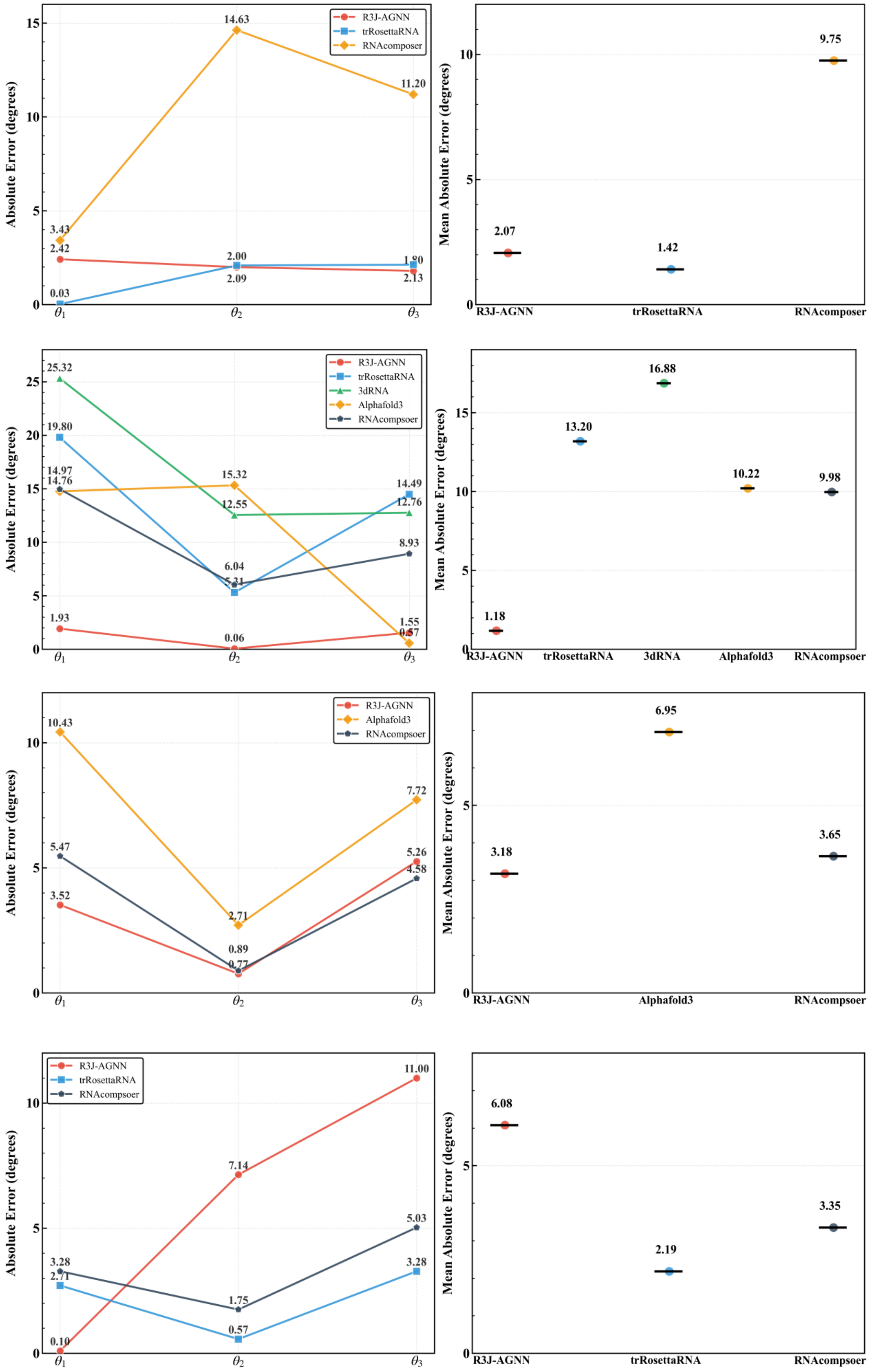

Figure S6. Geometric accuracy analysis for eight representative targets. Left panels display

per-angle absolute errors; right panels show the MAE. Comparisons are restricted to topologically verified methods. R3J-AGNN (red) consistently demonstrates superior stability across targets: 3PDR_A, 4QLM_A, 4R4V_A, 4WFL_A, 6P2H_A, 7QEP_C, 8FMW_Y, and 8RXH_H.

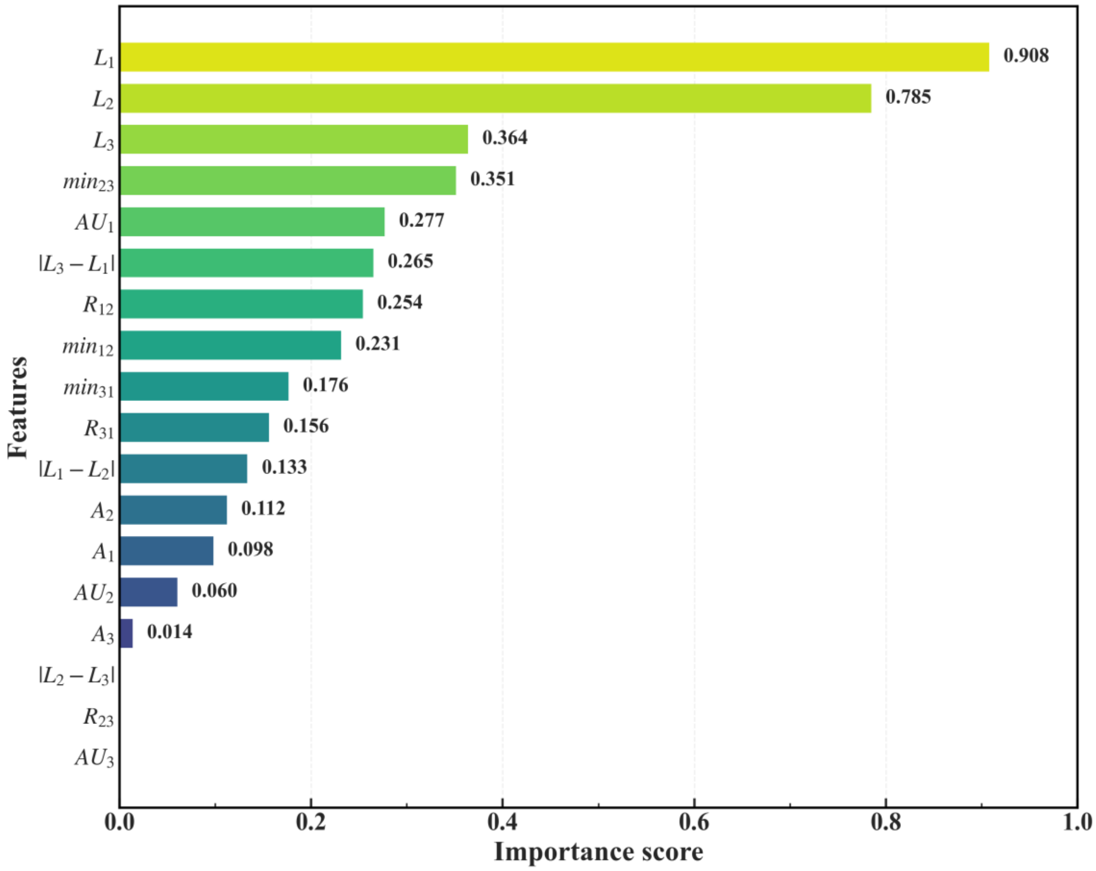

Figure S7. Permutation importance analysis of node features in the coarse-grained tree graph. Importance scores quantify the average increase in mean absolute error (MAE) after randomly permuting each feature group on the training set, thereby measuring its contribution to prediction performance.

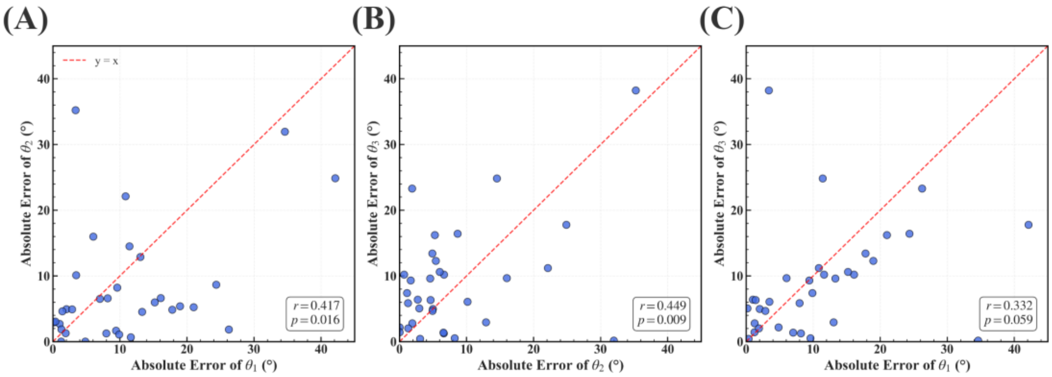

Figure S8. Pairwise correlation analysis of absolute prediction errors for the three inter-branch angles. Scatter plots display the relationship between the absolute angular errors of (A) $\Delta\theta_1$ versus $\Delta\theta_2$, (B) $\Delta\theta_2$ versus $\Delta\theta_3$, and (C) $\Delta\theta_3$ versus $\Delta\theta_1$. The red dashed line ($y = x$) indicates the axis of equal deviation. Pearson correlation coefficients (r) and corresponding p-values are shown for each pair.

(A) (B)

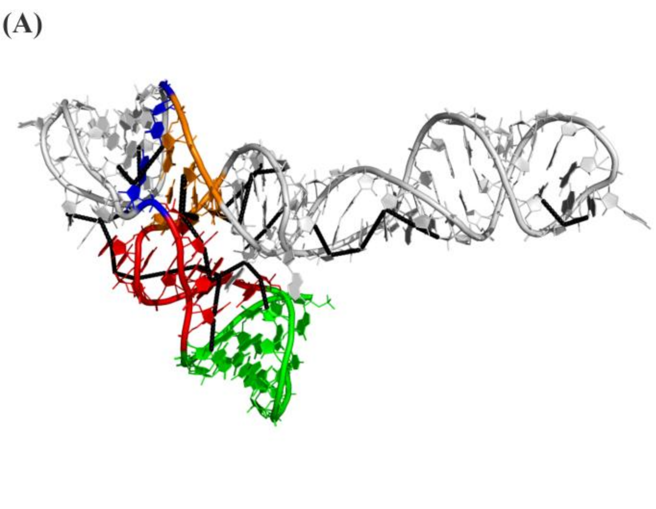

Figure S9. Visualization of tertiary interactions stabilizing RNA three-way junctions with coaxial-stacking. Representative 3D structures of (A) PDB ID: 3OXE (Chain A), and (B) PDB ID: 7R81(Chain C). The central three-way junction loop regions are highlighted in red. The three connecting stems, ordered from the 5' to 3' end, are colored green, blue, and orange, respectively. Black solid lines indicate spatial stabilizing forces, illustrating how local branch-loop base triples and long-range tertiary contacts cooperatively lock the coaxial stacking and the overall junction topology.
